# Randomized trial evaluating self-sampling for HPV DNA based tests for cervical cancer screening in Nigeria

**DOI:** 10.1186/s13027-017-0123-z

**Published:** 2017-02-06

**Authors:** Fatima Modibbo, K. C. Iregbu, James Okuma, Annemiek Leeman, Annemieke Kasius, Maurits de Koning, Wim Quint, Clement Adebamowo

**Affiliations:** 1 Department of Medical Microbiology and Parasitology, National Hospital Abuja, Plot 132 Central Business District (Phase II), PMB 425 Garki, Abuja, 90001 Nigeria; 2 Department of Research, Institute of Human Virology, Abuja, Nigeria; 3 DDL Diagnostic Laboratory, Visseringlaan 25, 2288 ER Rijswijk, Netherlands; 4 Department of Epidemiology and Public Health, University of Maryland School of Medicine, Baltimore, MD USA; 5 Institute of Human Virology and Greenebaum Comprehensive Cancer Centre, University of Maryland School of Medicine, Baltimore, MD USA

**Keywords:** Human papillomavirus, Self-sampling, Randomized trial, Cervical cancer, Screening

## Abstract

**Background:**

Cervical cancer incidence and mortality rates in Sub-Saharan Africa (SSA) remain high due to several factors including low levels of uptake of cervical cancer screening. Self-collection of cervicovaginal samples for HPV DNA testing may be an effective modality that can increase uptake of cervical cancer screening in SSA and hard to reach populations in developed countries. We investigated whether self-collection of cervicovaginal samples for HPV DNA tests would be associated with increased uptake of screening compared with clinic based collection of samples. Furthermore, we compared the quality of samples collected by both approaches for use in HPV genotyping.

**Methods:**

We conducted a community based randomized trial in a semi-urban district of Abuja, Nigeria with 400 women, aged 30 to 65 years randomized to either hospital-collection or self-collection of cervicovaginal samples. We compared cervical cancer screening uptake among the 2 groups and evaluated the concentration of human DNA in the samples by measuring RNase P gene levels using qPCR. High-risk HPV DNA detection and typing was done using the GP5+/6+ Luminex system.

**Results:**

Most participants in the self-collection arm (93%, 185/200) submitted their samples while only 56% (113/200) of those invited to the hospital for sample collection attended and were screened during the study period (*p* value < 0.001). Human genomic DNA was detected in all but five (1.7%) participants, all of whom were in the self-collection arm. The prevalence of high-risk HPV in the study population was 10% with types 35, 52 and 18 being the commonest.

**Conclusions:**

Our study shows that self-sampling significantly increased uptake of HPV DNA based test for cervical cancer screening in this population and the samples collected were adequate for HPV detection and genotyping. Cervical cancer screening programs that incorporate self-sampling and HPV DNA tests are feasible and may significantly improve uptake of cervical cancer screening in SSA.

## Background

Cervical cancer is the fourth most common cancer among women worldwide, with an estimated 528,000 new cases and 266,000 deaths in 2012 [1]. More than 85% of new cases occur in low and middle income countries (LMIC) and the incidence is projected to rise by 5% over the next 10 years [2]. It is the second most frequently diagnosed cancer and the leading cause of cancer deaths in Sub-Saharan African (SSA) women [2]. The Age-Standardised Incidence Rate (ASR) for cervical cancer in Nigeria was 34.5/100,000 population in 2012 [3].

The incidence and mortality of cervical cancer has declined significantly in developed countries due to widespread availability and uptake of cervical cancer screening [4]. These screening programs were initially based on cervical cytology but HPV DNA based tests are increasingly used. In contrast, cervical cancer screening in LMIC has not been so successful due to several factors including low levels of awareness, cost, cultural barriers and lack of screening programmes [5–7].

Several approaches have been investigated to provide cervical cancer screening in LMIC. Cytology based screening has not been effective therefore other methods such as visual inspection with acetic acid and HPV DNA testing are increasingly being evaluated [8, 9]. Studies have found that women screened for cervical cancer at least once in their lifetime using HPV DNA testing significantly reduce their risk of developing advanced cervical cancers and cervical cancer related deaths [8, 10].

Barriers to uptake of cervical cancer screening in LMIC such as cost, lack of infrastructure and concerns about health care workers’ attitudes can be overcome by using self-collection of cervico-vaginal samples paired with HPV DNA tests [11]. When combined with an effective means for sample transportation to the laboratory, return of results and prompt treatment, such screening methods may overcome many of the challenges associated with cervical cancer screening in LMIC [12]. Research into cost-effective cervical cancer screening methods that are based on HPV DNA tests which involve minimal number of clinic visits are urgently needed.

In this study, we compared the uptake of home based, self-collection with clinic based, health professionals’ collection of cervicovaginal samples for HPV DNA tests for cervical cancer screening in a semi-urban area in Abuja, Nigeria. We evaluated the quality of the samples collected to ascertain their utility of the HPV DNA tests and genotyped the HPV types found in this study population.

## Methods

### Setting and participants

We randomly selected Karu out of three semi-urban districts around Abuja, the capital of Nigeria, that had relatively heterogeneous populations based on socio-economic status, easy access to hospitals for health professionals collected cervicovaginal samples and a functioning postal system so that women who self-collect their samples can have the option of mailing it to our laboratory. Karu had a population of about 205,477 in 2006 [13].

#### Community engagement

We engaged the Karu community by meeting with the King of the community and his council members, explaining the study to them and seeking their permission to work in the community. After permission was granted, we met with key religious and opinion leaders, and organized a meeting with all members of the community at the King’s palace where we explained the rationale and methods of the study. We also visited all the religious and cultural gatherings that took place within the community during the study period and extended invitations to participate in the study to all women between 30 to 65 years of age.

### Enrollment and randomization

Between February 2014 and May 2014, all women interested in the study were invited to meet the research team at the King’s palace which is centrally located within the community. Inclusion criteria were women aged between 30 and 65 years, living or working in Karu who do not plan to move out of the community over the next 6 months. We excluded women who were pregnant, planning to relocate within six months, HIV positive, had unexplained cervical bleeding, history of hysterectomy, mental illness or cervical cancer from the study. All women provided written informed consent.

After consenting, all the women were given health education on cervical cancer, its risk factors, the research project, sampling procedure and randomization. We generated a random numbers’ list and created 2 groups. We assigned the 200 odd numbers on the list to the self-sampling group and the 200 even numbers to the hospital-based sampling group. Women were sequentially assigned to either the hospital-collection group or the self-collection group as they enrolled and consented to participate in the study. Women randomized to self-sample were given the self-sampling kit and instructions on how to use it while those assigned to the hospital group were given appointments for the clinic.

Participants were not blinded with regards to the intervention they received because this was not feasible.

### Study procedure

All the women enrolled in the study were administered a questionnaire in a language they understood by a trained research nurse. The questionnaire asked questions about socio-demographics, sexual health and behaviour, obstetrics and gynaecology history, vaginal health, as well as cervical cancer screening history. Measures incorporated in this study were selected from the PhenX Toolkit version July 31 2013, Ver 5.5.

### Sample collection

Women in the hospital-collected group had cervicovaginal samples collected at the National Hospital Abuja, Cervical Cancer Screening Clinic by trained nurses using a dry flocked swab (Copan Diagnostics INC CA USA).

Participants in the self-collection group used dry flocked swabs to collect cervicovaginal samples at home and inserted them into pre-stamped envelopes that we provided. They had the option to mail the envelope through the post-office, or drop the envelopes off at designated collection points within the community or at the National Hospital, Abuja, Nigeria.

We returned results of HPV DNA tests to participants’ via text messages on their cell phones and invited those who were positive for hrHPV to return for treatment and follow up.

### Laboratory analyses

The swabs were stored at −80°C at the Institute of Human Virology Nigeria Bioreprository prior to analyses. We performed the laboratory analysis at DDL Diagnostic Laboratory, Rijswijk, the Netherlands. Materials from swab specimens were suspended in 3mL of phosphate buffered saline (PBS). DNA was extracted from 750μl using the EasyMAG NucliSens extraction platform (BioMérieux, Boxtel, the Netherlands) [14]. The extracted DNA was eluted in 100μl of wash buffer 3. Each DNA extraction run contained positive and negative controls to monitor the extraction procedure. HPV detection and typing was performed using GP5+/6+ PCR-EIA system with LMNX genotyping according to the manufacturer’s instructions (LMNX Genotyping kit HPV GP HR, Labo Bio-medical Products, Rijswijk, the Netherlands) [15]. A 10μl aliquot of extracted DNA was used for each GP5+/6+ PCR. Quantitative real time PCR was performed to measure the human DNA concentration through detection of a 65bp fragment from the RNase P gene. Detection of the RNase P gene was used as a quality control measure for sample adequacy [16]. Five microliter of extracted DNA was added to 20 μl of PCR mastermix and amplification was performed on a Bio-Rad CFX96 real time PCR detection system (Bio-Rad Inc. Berkeley, CA, USA). Quantification of the amount of RNase P copies present in each sample was done by comparing the observed quantification cycle (Cq) of the sample to the Cq values of the standard curve with known concentrations of human DNA (i.e., 300000, 30000, 3000, 300 and 100 haploid genomic equivalent (GEQ) copies/PCR). Positive and negative PCR controls were used in each run. Samples were considered invalid if they had a negative RNase P result (Cq values of >40).

### Outcome measures

The primary outcome measure was uptake of cervical cancer screening using HPV DNA tests which we defined as the proportion of women who completed the screening procedure out of all women enrolled in each group during the study period. The study period lasted for one month after enrollments were completed. We also evaluated secondary outcomes including predictors of acceptance of screening and preference for self-sampling. We tested each swab for presence and type of high risk HPV and concentration of human DNA.

### Data analysis

We conducted intention-to-treat analysis. Women who responded to either method of screening (self-collection or hospital-collection) were considered as a positive result of the strategy to which they had been randomized regardless of which method they finally used for screening.

Study data were collected and managed using REDCap electronic data capture tools hosted at the Institute of Human Virology Nigeria [17]. We used logical checks and validation protocols to ensure high quality data. Statistical analysis of data obtained was performed using STATA version 12 software (StataCorp, College Station, Texas, USA). Means and standard deviations were used to describe continuous variables while categorical variables were expressed in terms of frequencies and proportions. We computed socioeconomic status using Principal Component Analysis of Wealth Index data as described [18].

Logistic regression models were used to evaluate predictors of screening uptake including baseline characteristics and prior knowledge of cervical cancer. Odds ratios (OR) and 95% confidence intervals (CI) were generated. An association was statistically significant if the *p-*value was less than or equal to 0.05. We used differences in mean human DNA concentrations to determine efficiency of swabs collected using student-t tests. Samples with absent RNase P (Cq values >40) were excluded from the data analysis.

We obtained ethical approval for the study from the National Hospital Abuja, Health Research Ethics Committee (Approval number NHA/EC/238/20).

### Role of the funding source

The funder had no role in study design, data collection, data analysis, data interpretation, or writing of the report. The corresponding author had full access to all data used in this study and had final responsibility on the decision to submit for publication.

## Results

**Fig. 1:**
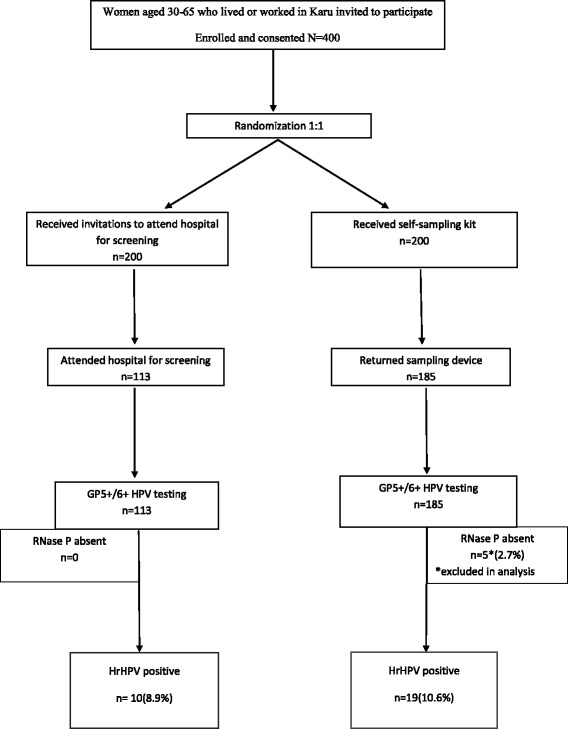
Flow chart showing enrolment, randomization and testing results

We enrolled 400 women who consented into the study. Figure 1 shows the study design and the women who participated in the study. The mean (SD) age of the women was 40.8 (1.3) years. Most participants (337, 84.3%) were in the age group 30–49 years, 328 (82%) were married and 331 (82.8%) were Christians. About half (199, 49.8%) of the participants had attended tertiary level of education and 212 (53%) belonged to the middle socioeconomic class. Some 47% (188/400) of women enrolled in the study had heard of cervical cancer (Table 1). Of this number, 45% heard about the disease from the television, 22.5% from personal communications, 17.6% from a health care practitioner, 17.1% from other forms of mass media and 8% from church awareness programs. Most of the participants, 98.8% (395/400) had never been screened for cervical cancer and the reasons for this included lack of access to screening services (49.7%), lack of time (8.7%), financial constraints (7.2%), lack of knowledge of screening (6.2%) and long hospital waiting times (3.6%).

**Table 1:** Baseline Characteristics for Women Enrolled in Study *N =* 400

	Hospital-collected (*N =* 200)	Self-collected (*N =* 200)	All (*N =* 400)	*P* value
Mean age in years (SD)	40.3(0.99)	41.3(1.06)	40.8(1.29)	
Age in years				0.132
30–39	103(51.5%)	82(41%)	185(46.3%)	
40–49	67(33.5%)	85(42.5%)	152(38%)	
50–59	26(13%)	31(15.5%)	57(14.3%)	
60 and above	4(2%)	2(1%)	6(1.5%)	
Religion				0.597
Christianity	163(81.5%)	168(84%)	331(82.8%)	
Islam	37(18.5%)	32(16%)	69(17.3%)	
Marital status				0.152
Married	158(79%)	170(85%)	328(82%)	
Not Married	42(21%)	30(15%)	72(18%)	
Educational status				0.592
No formal schooling	19(9.5%)	16(8%)	35(8.8%)	
Primary education	38(19%)	30(15%)	68(17%)	
Secondary education	35(17.5%)	41(20.5%)	76(19%)	
Tertiary education	96(48%)	103(51.5%)	199(49.8%)	
Socioeconomic status				0.484
Upper class	14(7%)	12(6%)	26(6.5%)	
Middle class	100(50%)	112(56%)	212(53%)	
Lower class	86(43%)	76(38%)	162(40.5%)	
Heard of cervical cancer				0.483
Yes	90(45%)	98(49%)	188(47%)	
No	110(55%)	102(51%)	212(53%)	
Ever screened for cervical cancer				1.000
Yes	2(1%)	3(1.5%)	5(1.3%)	
No	198(99%)	197(98.5%)	395(98.8%)	
No of sexual partners in the last 12months				0.286
1	163(81.5%)	169(84.5%)	395(98.8%)	
2–3	5(2.5%)	2(1%)	7(1.8%)	
≥ 4	0(0%)	2(1%)	2(0.5%)	
Unknown	32(16%)	27(13.5%)	59(14.8%)	
Age at sex initiation				0.150
< 16	27(13.5%)	15(7.5%)	42(10.5%)	
16–19	66(33%)	61(30.5%)	127(31.8%)	
≥ 20	88(44%)	106(53%)	194(48.5%)	
Unknown	19(9.5%)	18(9%)	37(9.3%)	

Overall 75% (298/400) of the women completed cervical cancer screening within the study period. Most of the women in self-collection arm, 93% (185/200), were screened while 56% (113/200) of those invited to hospital completed screening during the study period (*p* < 0.001). All 185 (100%) self-collected swabs were submitted within 24 h of cervicovaginal sample collection in a designated collection box in the community. Most of the women in the self-collection arm of the study chose this option because they found it convenient (84.3%, 156/185) while 10.3% (19/185) chose this option because they did not trust the postal service to deliver the swabs in a safe and timely manner. Most of these women (95.2%, 177/185) found the self-sampling device easy to use while 4.3% (8/185) found it difficult to use and 83.2% (154/185) would prefer self-sampling as a future screening option than hospital-sampling (Table 2). There was no association between age, religion, marital status, awareness of cervical cancer, education, socio-economic status, and uptake of screening (Table 3).

**Table 2:** Operational Aspects of Cervicovaginal Self-collection *N =* 185

Variable	n	%	95% CI
Reason for preference of collection box			
Convenient	156	84.3	79.6;90.0
Distrust of postal service	19	10.3	5.9;14.6
Privacy	5	2.7	0.4;5.0
Cultural reasons	5	2.7	0.4;5.0
Sample device			
Easy to use	177	95.7	92.1;98.3
Difficult to use	8	4.3	1.4;7.2
Future screening preference			
Self-sampling	154	83.2	77.8;88.6
Comfortable	134/154	87.0	
Private	10/154	6.5	
Less embarrassing	5/154	3.3	
To ensure the right sample is taken	3/154	2.0	
Financially convenient	1/154	0.6	
Sense of independence	1/154	0.6	
Hospital-sampling	17	9.2	5.0;13.4
To ensure right sample is taken	13/17	76.5	
Comfortable	3/17	17.7	
Better option	1/17	5.8	
No Preference	14	7.6	3.8;11.4

*CI* Confidence intervals

**Table 3:** Predictors for Screening Uptake

Predictor	Adjusted OR^a^	95% CI^a^	*P* value^a^
Age			
30–39	Reference		
40–49	1.40	0.80–2.44	0.237
50 above	1.08	0.53–2.31	0.835
Religion			
Christianity	Reference		
Islam	1.49	0.76–2.93	0.247
Marital status			
Not Married	Reference		
Married	1.15	0.62–2.14	0.651
Education			
No formal schooling	Reference		
Formal schooling	1.06	0.54–2.20	0.861
Socioeconomic status			
Lower class	Reference		
Middle class	1.14	0.68–1.91	0.627
Upper class	1.07	0.38–2.99	0.894
Cervical cancer awareness			
No	Reference		
Yes	0.76	0.48–1.20	0.234

*OR* Odds Ratio, *CI* Confidence intervals

^a^OR, 95% CI and P values obtained from logistic models adjusting for method of sample collection

The median (IQR) of DNA concentrations from the self-collected (*n* = 185) and hospital-collected (*n* = 113) swabs were 10.5 (2.81–27.68) and 6.6 (2.37–11.80) respectively. There was a significant difference in DNA concentration between self-collected and hospital-collected samples (*p* = 0.003).

Of the 298 samples received, 29 (10%) were positive for hrHPV. The prevalence of hrHPV infection among women in the self-collection group was 8.9% (19/185) while it was 10.3% (10/113) among women in the health professionals’ collection group. These were not statistically significantly different (*p* = 0.84). The prevalence of hrHPV infections by age groups were 8.5% for women in the 30–39 years’ age group, 12.4% for those in 40–49 years’ age group and 6.4% for those aged 50 years and above. Among the 29 women positive for hrHPV, the mean (SD) age at sexual debut was 20.2 (6.4) years compared with 20.3 (4.3) years among the hrHPV negative women. There were no associations between hrHPV positivity and either socio-economic status (*p* = 0.29) or obesity (*p* = 0.69). Age, religion, marital status, hormonal contraceptive use, and douching were not significantly associated with risk of hrHPV infection. None of the hrHPV positive women smoked cigarettes and 89.7% (26/29) reported having only one sexual partner in the last 12 months.

**Fig. 2:**
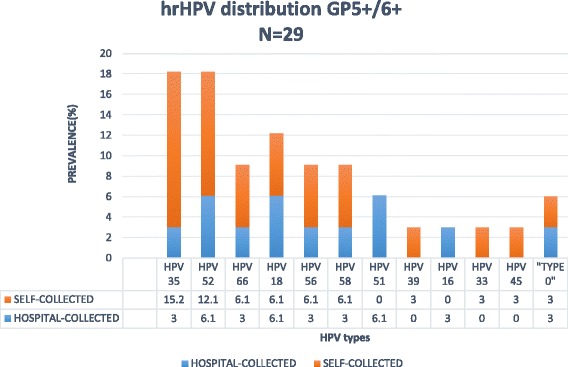
High risk HPV distribution based on clinically validated GP5+/6+ assay

Most of the women with hrHPV infection (24, 82.8%) had single hrHPV infections spanning 11 types (Fig. 2), 3 (10.3%) had multiple hrHPV infections viz. 16 and 18, 35 and 56, and 35, 56 and 58 while 2 infections (6.1%) were not classifiable and were designated “Type X” (Fig. 2). Types 35 (18.2%), 52 (18.2%) and 18 (12.2%) were the most prevalent hrHPV types in this study. Types 16 was found in only one (3.0%) participant.

## Discussion

In this study, we found that significantly higher proportion of women in the self-collection group completed HPV DNA based tests for cervical cancer screening compared to women invited to hospital for health professionals’ collections of samples. Our results show that self-collection is a viable method of increasing cervical cancer screening uptake in LMIC. Similarly high response rates for self-sampling have been reported in Uganda and Cameroun [19, 20]. In developed countries where organized screening programs are available, self-sampling has been shown to improve cervical cancer screening rates among women who fail to attend regular screening [21]. Although women in the self-sampling arm of this study had several options for sending their samples to the lab, all participants chose to leave their samples at designated collection points in the community. This result was not unexpected given the poor quality of mail services in Nigeria. Concerns about sending such an intimate biological sample through the postal system may also have influenced this choice.

Majority of the women in the self-sampling group would prefer to repeat self-sampling at their next screening visit instead of having to visit a hospital. This is similar to findings from other self-sampling studies in low and middle income countries [19, 22]. In Kenya, lack of transportation, cost and long hospital queues were reported as deterrents to hospital-based screening [23]. Majority of participants in this study identified lack of cervical cancer screening services in healthcare facilities as the reason why they had not participated in screening in the past. Given the high morbidity and mortality of cervical cancer, and the availability of screening options, research into methods of implementation of cervical cancer screening that would have high levels of uptake in the community is urgently needed. Other important barriers to cervical cancer screening in Nigerian have been previously published [11].

None of the characteristics of participants evaluated were found to be significantly associated with screening uptake in this population. This finding strengthens the argument that the major deterrent to cervical cancer screening in LMIC is non-availability. Most SSA countries lack of human resources and infrastructure required for establishment of systematic cervical cancer screening, and their health budgets need to address competing needs from infectious diseases [24]. To be successful, screening programs in SSA should to be based on more abundant cadre of health professionals like nurses, midwives and community health workers [9].

Almost all our participants found the dry flocked swab easy to use. This finding is comparable to that from studies in other SSA countries where similar collection device was used [20, 25, 26]. Previous studies have documented that women were concerned about their ability to successfully sample themselves [19, 27]. Our study shows that this concern can be assuaged by properly educating the women on how to perform the procedure prior to collection [20].

All women in the self-sampling group returned their swabs within 24 h of collection. We found that most samples except five from self-collection group had adequate DNA samples based on RNase P levels. The use of RNase P to check adequacy of human genomic DNA present in biological samples is well established [16]. This finding reinforces the evidence that women can be educated to collect samples of adequate quality. Studies of self-collected samples that are processed after longer intervals between collection and analyses should be conducted in order to evaluate whether this approach is robust in the health care settings.

The prevalence of hrHPV infection in this study is similar to findings from our previous studies of women who presented for screening at hospitals in Abuja, Nigeria [28, 29]. This suggests that the population point prevalence of hrHPV in Nigeria is 10%. We detected eleven types of hrHPV in this study with types 35, 52 and 18 predominating and constituting almost 50% of all types detected. HPV 35 has been reported as the commonest type found in women with normal cytology in Sub-Saharan Africa [30], and studies conducted in Benin Republic, Guinea, Mozambique and Abuja have all published similar findings [28, 31–33]. The finding of HPV 52 and 18 being among the most prevalent types is consistent with other Nigerian studies [34, 35]. These three types of HPV are among eight that account for 86% of cervical cancers worldwide [36]. Although HPV 16/18 infections account for majority of the disease worldwide, the contributions of HPV 16 to invasive cervical cancer from Sub-Saharan Africa and in particular West Africa is among the lowest globally [36, 37].

A limitation of our study is the use of interviewer administered questionnaires which may have skewed responses to some of the sensitive questions towards what was perceived to be more socially acceptable, however studies have shown that the influence of biased responses are minor and do not affect overall results [38, 39]. The demographic characteristics of our participants also differs from that of the general Nigeria population and this may limit the generalizability of our results [40]. Some members of the community may have opted not to respond to the invitations to participate in the study and we cannot rule out healthy volunteer bias.

## Conclusions

Our findings further strengthens the evidence that cervicovaginal self-sampling is an acceptable modality that can lead to a substantial increase in the proportion of women who are screened for cervical cancer in LMIC. The quality of cervicovaginal samples collected by self-sampling is adequate for HPV DNA detection and typing. This justifies further studies that integrate this modality into cervical cancer screening programs in LMIC.
